# Inflammation—A Link Between Arterial Atherosclerotic and Venous Thromboembolic Diseases

**DOI:** 10.3390/cells14171319

**Published:** 2025-08-27

**Authors:** Pavel Poredos, Peter Poredos

**Affiliations:** 1 Department of Vascular Disease, University Medical Centre Ljubljana, Zaloska 7, 1000 Ljubljana, Slovenia; pavel.poredos@kclj.si; 2 The Faculty of Medicine, University of Ljubljana, Vrazov trg 2, 1000 Ljubljana, Slovenia; 3 Department of Anaesthesiology and Surgical Intensive Care, University Medical Centre Ljubljana, Zaloska 7, 1000 Ljubljana, Slovenia

**Keywords:** arterial atherosclerotic disease, venous thromboembolism, deep venous thrombosis, inflammation, interleukins, anti-inflammatory drugs

## Abstract

An increasing body of evidence suggests the likelihood of a link between arterial atherosclerotic disease (AAD) and venous thromboembolic disease (VTED). Inflammation is accepted as a basic pathogenetic mechanism of both diseases. The involvement of inflammation in the pathogenesis of AAD and VTED is supported by increased levels of circulating inflammatory markers, particularly interleukins, which are involved in the development and progression of atherosclerosis as well as in thrombus formation in arterial and venous beds. A consideration supporting a close link between these diseases is also based on the evidence of common risk factors which promote the development of both diseases through stimulation of systemic inflammation. Further, the relationship between arterial and VTED is supported by findings of the simultaneous appearance of clinical or preclinical AAD and VTED. The aim of this narrative review is to report evidence of the inflammatory basis of arterial and venous diseases, which is important for common therapeutic procedures. Besides classical drugs used in the prevention of arterial and venous diseases with their pleotropic anti-inflammatory activity, new anti-inflammatory drugs provide the possibility for treatment of both AAD and VTED and could represent a unified therapeutic approach to both diseases.

## 1. Introduction

Arterial atherosclerotic disease (AAD) and venous thromboembolic disease (VTED) represent the leading causes of morbidity and mortality in developed countries [1]. AAD is the most common underlying cause of ischemic heart disease, stroke, and peripheral arterial disease (PAD), and deep venous thrombosis and pulmonary embolism constitute VTEDs [2]. In both diseases, thrombus formation with occlusion of vessels is a final event. Arterial thrombosis is primarily driven by atherosclerotic plaque rupture and platelet aggregation, while venous thrombosis is linked to stasis and hypercoagulability. As arterial thrombosis is largely a phenomenon of platelet activation, it is defined as a white thrombus, whereas venous thrombosis is predominately associated with activation of the coagulation system and is defined as a red thrombus. Therefore, until recently, the link between these two diseases was overlooked. However, recent studies have shown that this dichotomy is likely to be an oversimplification. Accumulating evidence suggests that they share common basic pathogenetic mechanisms and risk factors. The most evident indicator of an interrelationship between AAD and VTED is inflammation as a basic pathogenetic mechanism of both diseases. Inflammation is a common mechanism through which different risk factors trigger damage to vessel walls and promote thrombus formation in both veins and arteries. Inflammation of a vessel wall is represented by activated monocytes and macrophages, which enter the subintimal vessel space and initiate damage to arterial or venous walls. This causes atherosclerosis or destruction of the venous wall structure, which predisposes to thrombus formation [3]. In the case of atherosclerosis, the most common initiating factor which damages vessel walls is the accumulation of LDL cholesterol, while venous wall damage is primarily affected by venous stasis or is the consequence of a systemic inflammatory process caused by inflammatory disease [4]. Inflammation provokes endothelial dysfunction, which is a systemic disorder simultaneously affecting arterial as well as venous systems. Endothelial dysfunction causes disbalance between prothrombotic and anti-thrombotic factors, leading to a predisposition to thrombus formation [5].

This relationship is also indicated by the effect of drugs used for the management of both diseases. While the prevention and treatment of AAD are now mostly based on platelet inhibition and elimination of risk factors, and VTED is managed with anticoagulants, recent findings indicate that anticoagulants are also effective for the prevention of atherosclerotic cardiovascular events [6], and antiplatelets exert some effects in the prevention of VTED.

Further, there is considerable evidence supporting a close link between these two diseases based on common risk factors for arterial and venous thrombotic diseases, which are more common than previously realized [7]. This interrelationship is also supported by clinical findings. Individuals that suffer from idiopathic VTED are at markedly increased risk of suffering a cardiovascular event and also have an increased incidence of AAD [8]. Therefore, recent findings indicate that arterial and venous thromboembolic diseases represent a different presentation of the same or similar disease.

The aim of this narrative review is to elucidate the role of inflammation as a common basic pathogenetic mechanism of AAD as well as VTED and to discuss the efficacy of similar preventive measures in both diseases.

## 2. Relationship Between Inflammatory Markers and Peripheral Arterial and Venous Thromboembolic Diseases

Arterial atherosclerotic disease: Increased levels of inflammatory biomarkers were found in patients with PAD and other AADs [9]. Patients with preclinical or clinical AAD have increased circulatory inflammatory markers, which are associated with the development and progression of PAD [10]. The Edinburgh Artery Study showed that C-reactive protein (CRP), interleukin-6 (IL-6), and soluble and adhesion molecules are predictors of progressive peripheral atherosclerosis [11]. Among inflammatory markers, the pro-inflammatory cytokine IL-6 was shown to be the strongest predictor of PAD and was independently associated with disease progression [11]. Also, other interleukins, E-selectin, and metalloproteinases are involved in the pathogenesis of PAD and predict major cardiovascular (CV) events in patients with severe limb ischemia [12]. In the Atherosclerotic Risk in Communities (ARIC) study, monocyte chemoattractant protein-1 (MCP-1) was associated with the progression of PAD and inversely correlated with ankle–brachial index (ABI) [13]. Similarly, serum levels of β2-microglobulin were also associated with ABI [14]. As atherosclerosis is a systemic disease, it is to be expected that in different AADs there are similar or identical circulating markers. However, there are some differences in biomarkers between coronary heart disease (CHD) and PAD, which are caused by different endothelial and smooth vascular muscle gene expression in different vascular beds [15].

Venous thromboembolic disease: Recently, inflammation has been accepted as a possible mechanism through which different risk factors trigger thrombus formation in veins also [16]. Inflammation and hemostasis are coupled by common activation pathways and feedback regulation systems. Inflammation results in the increased production of procoagulant factors and in the downregulation of anticoagulant mechanisms [17]. Several studies have indicated a predictive value of certain inflammatory markers for the development of VTED. The most frequently studied markers are as follows: CRP, inflammatory cytokines, MCP-1, tumor necrosis factor alpha (TNF–α), and others.

C-reactive protein: The predictive value of CRP has been examined in large prospective studies, but the results are contradictory. In the Physician Health Study, 22,071 male US physicians were followed for up to 14 years. The mean CRP plasma levels in subjects who developed VTED during the observational period were not statistically different from subjects who did not develop VTED [18]. Similarly, the Cardiovascular Health Study and the Atherosclerosis Risk in Communities study also demonstrated that there was no association between baseline CRP levels and subsequent development of VTED [19].

In one of our studies, patients with idiopathic deep venous thrombosis (DVT) had increased levels of inflammatory markers 2–4 months after an acute event, including CRP [20]. The systemic review and meta-analysis included 65,162 participants, of whom 1289 developed VTED during long-term observation (up to 16 years) (VTED patients with transient risk factors were excluded), and showed that levels of CRP and high-sensitivity CRP (hs-CRP) might be regarded as risk factors for future VTED occurrence [21]. Similarly, Folson et al., in a multivariable analysis in the Atherosclerosis Risk in Communities (ARIC) cohort study, demonstrated that CRP was associated with VTED appearance [22]. A meta-analysis confirmed an increased risk of VTED in patients with increased CRP, and a linear dose–response relationship was shown [23]. In contrast, the Tromso study concluded that hsCRP is not a risk factor for VTED. Some studies also evaluated CRP as a diagnostic tool for the detection or exclusion of VTED. But several larger studies concluded that CRP, because of its low specificity, does not have utility for rolling-in or rolling-out DVT in patients with clinically suspected DVT.

Interleukins play a significant role in the development of VTED. Cytokines are multifunctional endogenous mediators of the inflammatory response, leading to thrombus formation or resolution [24]. Elevated levels of certain interleukins, particularly IL-6 and IL-8, have been associated with an increased risk of VTED. Increased levels of IL-8 have been found in patients experiencing a first event of DVT and in patients with recurrent thrombosis [25]. The Leiden Thrombophilia Study showed that patients with increased levels of IL-8 had a twofold increased risk of VTED, and the risk seemed to increase with the actual level of IL-8 [26]. Several studies have also shown an association between plasma levels of IL-6, VTED, and post-thrombotic syndrome [27]. Wojcik et al. demonstrated that IL-6 is a potential target for post-thrombotic syndrome, which represents a frequent complication of VTED [28]. The role of IL-6 in the pathogenesis of VTED is also indicated by the beneficial effect of IL-6 neutralization, which reduces inflammation during thrombus formation and fibrosis that occurs after thrombus resolution [29]. Studies have also shown increased IL-6 expression in DVT patients [30]. Further, upstream regulatory factors leading to increased interleukin expression have been identified as risk factors of VTED. Additionally, it was shown that micro ribonucleic acids (microRNAs) play an important role as suppressors of IL-6, and decreased microRNA levels increase IL-6 expression and promote DVT formation [29]. In the Leiden Thrombophilia Study (LETS), increased levels of cytokines (IL-6, IL-8, and TNF-alpha) in the prethrombotic period were associated with a 2–3-fold increased risk of a first episode of DVT [26].

Other interleukins, such as IL-10 and TNF-alpha, appear to promote thrombus resolution. Interleukin-10 is a cytokine exerting anti-inflammatory properties and maintaining normal homeostasis [31]. Downing et al. demonstrated in a rat model that IL-10 neutralization resulted in heightened vein wall inflammation, revealed by leukocyte infiltration [32]. One of our studies showed that patients with idiopathic DVT have significantly decreased levels of IL-10 and increased levels of pro-inflammatory cytokines [33]. This imbalance between pro- and anti-inflammatory cytokines indicates that inflammation is involved in the etiopathogenesis of VTED.

Further, other interleukins, such as IL-17, IL-9, IL-1β, and TNF-α, may be involved in thrombus formation and resolution. However, their definite role in the pathogenesis of VTED has to be elucidated in large control studies.

There is no definite answer as to whether increased inflammatory markers are a cause or a consequence of VTED. Most of the studies mentioned investigated the relationship between inflammatory markers and VTED during the acute phase. The study of Jezovnik et al. investigated circulating levels of inflammatory markers in patients with a history of DVT 5 years after an acute event. Patients with idiopathic DVT had long-term increased inflammatory markers. This finding favors the hypothesis that inflammation is a cause and not merely a consequence of acute VTED [34].

In conclusion, inflammatory markers play a significant role in both AAD and VTED. Common inflammatory markers include CRP, interleukins (especially IL-6 and IL-8), TNF-alpha, and E-selectin. These markers indicate the presence and the extent of inflammation, which is a key factor in the development and progression of both diseases. On the other hand, anti-inflammatory interleukins, particularly IL-10, are decreased in AAD as well as in VTED.

## 3. Common Risk Factors of Vascular Disease and Inflammation

AAD and VTED are interrelated through common risk factors which provoke systemic inflammatory responses. The risk factors, reported to be common in both arterial and venous diseases, include increasing age, overweight, smoking, exposure to estrogens, and the presence of diabetes [7]. Further, dyslipidemia (increased low-density lipoprotein (LDL) and decreased high-density lipoprotein (HDL) cholesterol), which is a classical risk factor of AAD, also represents a risk for VTED [35,36].

Smoking is a well-established risk factor for AAD and is associated with increased risk of VTED [37]. Tobacco smoke initiates a substantial inflammatory response, which leads to the dysfunction of cells regulating immunity [38]. Further, smoking provokes a rise in neutrophil and circulating T-cell counts and their activity [39].

Obesity: Adipose tissue releases a variety of inflammatory mediators, such as interleukins (IL-1, IL-6) and TNF-α [40]. Visceral fat in particular is associated with increased levels of circulating biomarkers of inflammation [41]. The production of inflammatory mediators by adipose tissue has been considered an important mechanism for the promotion of atherosclerosis development. However, obesity is also an independent risk factor for VTED, and the risk increases with increasing body mass index (BMI) [42].

Dyslipidemia: LDL cholesterol has prothrombotic and endothelium-deteriorating effects, which are not limited to the arterial system. The association between hypercholesterolemia and VTED has been established [43]. Dyslipidemia promotes inflammation as a common pathogenetic mechanism of both diseases through several mechanisms. It inhibits hormone-sensitive lipase that hydrolyzes triglycerides within adipocytes and releases free fatty acids [44]. Circulating free fatty acids promote inflammation by activating Toll-like receptor-2 on the cell surface [45], which damages arterial as well as venous walls.

Hypertension: Increasing evidence indicates that inflammation is involved in the genesis and evolution of hypertension. The elevation of blood pressure is the consequence of vascular inflammation and microvascular remodeling [41]. However, hypertension by itself provokes a chronic inflammatory response, based on the accumulation and activation of inflammatory cells and the release of pro-inflammatory cytokines and free radicals [46]. Therefore, increased blood pressure, in addition to mechanical deterioration of vessel walls, also directly promotes inflammation of arterial as well as venous walls. Hypertension is a well-known classical risk factor of AAD; however, its role in the pathogenesis of VTED has not been definitely elucidated. A cohort analysis of 5.5 million UK adults and mendelian randomization studies indicated that low blood pressure is associated with an increased risk of VTED [47]. In contrast to these findings, some studies indicated that hypertension could contribute to a prothrombotic state and increase the risk of blood clot formation in veins [48]. In the Nurses’ Health Study, hypertension was associated with a twofold increase in the risk of unprovoked pulmonary embolism [49].

Diabetes mellitus promotes inflammation of vessel walls through insulin resistance, hyperglycemia, dyslipidemia, and oxidative stress. Patients with type 2 diabetes mellitus have higher systemic inflammatory markers, such as high-sensitivity C-reactive protein (hs-CRP) and interleukins. Further, hyperglycemia promotes reactive oxygen species formation, which stimulates the expression and release of inflammatory and adhesion molecules. Therefore, diabetes mellitus promotes the development of diabetic atherosclerosis through increased systemic inflammatory responses [50]. In addition, diabetes mellitus has been shown to be associated with significant increased risk of deep venous thrombosis and pulmonary embolism. A Korean case–control study demonstrated that both low levels of HDL cholesterol and elevated fasting glucose correlated with a twofold increased risk of VTED [51].

Sedentary lifestyle is a known risk factor of atherosclerosis [52] and is associated with high risk of VTED [53]. One study showed that longer sedentary time was related to higher levels of IL-6 [54], which indicates a systemic inflammatory response involved in arterial as well as venous thromboembolic diseases.

The lack of a statistically significant influence of levels of inflammatory markers on the development of VTED in some studies might be explained by the different pathogenetic mechanisms of VTED. Some studies included patients with provoked and unprovoked VTED. In provoked VTED, there is a transient factor which causes damage to venous walls, followed by an inflammatory response which is usually short-lived. In unprovoked VTED, inflammation is most probably the primary and basic pathogenetic mechanism which provokes inflammation of venous walls, which is followed by thrombus formation. Therefore, it is expected that in unprovoked VTED, inflammatory markers including CRP are already increased in the prethrombotic period and permanently represent a risk of VTED.

## 4. Association Between Preclinical or Clinical Atherosclerosis and Venous Thromboembolic Disease

Numerous studies have indicated the relationship between clinical or preclinical AAD and VTED, particularly DVT. In a retrospective cohort study which included 151 patients with a history of spontaneous VTED and 151 control subjects, the rates of subsequent arterial events (acute myocardial infarction (AMI), ischemic stroke, and PAD) were studied [55]. During a follow-up of 43 months, there were significantly more CV events in patients with VTED compared to the control group (16 vs. 6). The difference remained significant after adjusting for age and other CV risk factors. Meanwhile, in the study from Grady et al., women with a history of myocardial infarction had a 2.1-fold higher risk for VTED over the entire course of the follow-up, while during the first 90 days after infarction, the risk was higher than fivefold [56].

Further, growing evidence indicates that VTED increases the risk of subsequent arterial thromboembolic events, primarily myocardial infarction (MI) or ischemic stroke [57,58]. Sornsen et al. published the results of a retrospective analysis of Danish medical databases involving more than 40,000 patients with VTED and 160,000 controls, followed for 20 years. Patients with DVT during the first year of the acute event had a relative risk for MI of 1.60 and for stroke of 2.19 [59]. Increased risks of MI and stroke were also observed during the subsequent 20 years of follow-up. The association between VTED and AAD was also investigated in the Tromso study, which included 82,000 subjects without previous VTED or AAD [60]. During the median follow-up of 12.2 years, subjects suffering from venous thromboembolic events had a 35% higher risk of future arterial events. In a large longitudinal cohort study of the medical records of patients participating in the ABI Study at the Mayo Clinic from 1996 to 2020, which included 39,834 subjects, the relationship between ABI (as a measure of PAD) and VTED was studied. After risk factor adjustment, the risk for VTED was modestly increased in PAD, and the greatest risk was seen in patients with severely low ABI [61].

The link between AAD and VTED is also supported by the findings of a relationship between preclinical AAD and VTED. Prandoni first showed that there is a link between preclinical atherosclerotic lesions and VTED [62]. In this study, patients with unprovoked DVT had significantly higher numbers of carotid atherosclerotic plaques than patients with provoked DVT or control subjects. It was concluded that there is an association between AAD and spontaneous DVT and that atherosclerosis may induce DVT, or that the two conditions may share common risk factors. Our previous studies showed that patients with a previous history of unprovoked DVT have a significantly higher intima–media thickness of carotid and femoral arteries and a higher prevalence of atherosclerotic plaques than controls. Also, the number of arterial segments involved and total plaque thickness were significantly higher in patients in comparison to controls [63]. The association between these two vascular diseases was also indicated by the impaired flow-mediated vasodilatory response of the brachial artery, as an indicator of endothelial dysfunction [64]. These results indicate the involvement of the functional deterioration of vessel walls, which affects both arteries and veins, and is probably involved in the pathogenesis of unprovoked DVT as well as atherosclerosis through inflammation.

In conclusion, several findings support the interrelationship between clinical and preclinical manifestations of AAD and VTED, particularly unprovoked DVT, and indicate that patients suffering from VTED have a heightened risk of various AADs. This relationship assumes a common pathogenetic mechanism—inflammation (Table 1).

## 5. Anti-Inflammatory Therapies for Vascular Disease

As inflammation represents a common pathogenetic mechanism of both AAD and VTED, similar or identical therapeutic options are expected. Patients with AAD should receive lipid-lowering drugs and other therapies to reduce the risk of CV events. However, despite multiple preventive and therapeutic options, residual inflammatory risk remains. Although it has long been known that atherosclerosis is a chronic inflammatory disease, little attention has been given to anti-inflammatory treatments of atherosclerosis.

Low doses of antiplatelet agents, which also exhibit anti-inflammatory activity, are prescribed to patients at risk for atherosclerosis, but the main focus for the prescription of these drugs has been their antiplatelet and antithrombotic activity and not their anti-inflammatory effect. Recently, anti-inflammatory effects have been recognized in some drugs that primarily do not possess an anti-inflammatory activity, such as statins. The JUPITER trial (Justification for the Use of Statins in Primary Prevention: An Intervention Trial Evaluating Rosuvastatin trial) has shown that the preventive effects of statins are linearly associated with their anti-inflammatory effects [65]. Therefore, statins are also effective in subjects who have normal LDL cholesterol levels but increased hs-CRP levels.

Recently, new anti-inflammatory drugs which directly inhibit inflammatory processes in patients with AAD are being investigated. Low-dose colchicine represents an anti-inflammatory treatment option for patients with stable atherosclerosis. The COLCOT (Colhicine Cardiovascular Outcomes Trial) study showed that colchicine lowered major CV events by 31% among patients with stable atherosclerosis [67]. Inhibition of interleukin 1-beta in the CANTOS (Canakinumab Anti-inflammatory Thrombosis Outcomes Study) trial showed the utility of targeting inflammation with interleukin inhibitors in chronic atherosclerosis [68].

Despite the growing evidence of the involvement of inflammation in the pathogenesis of VTED, the importance of anti-inflammatory treatment of this disease has been overlooked. For the acute treatment of DVT, anticoagulant drugs have been proven to be more effective than aspirin. On the other hand, several studies have shown that aspirin, with its antiplatelet and anti-inflammatory effects, may reduce DVT recurrence. Aspirin after anticoagulant treatment reduced the overall risk of recurrence by more than a third in a broad cross-section of patients with a first episode of unprovoked VTED [66]. There is also evidence that statins, the basic anti-atherosclerotic drugs, may reduce the risk of VTED [36]. Furthermore, the results of two meta-analyses have pointed to the effectiveness of statins in the prevention of VTED, particularly in subjects with high hs-CRP and normal lipid values [69,70].

Therefore, recognizing the inflammatory basis of arterial and venous diseases is important from a common therapeutic point of view. New drugs with anti-inflammatory effects hold promise for the treatment of both AAD and VTED and could represent a unified therapeutic approach to both diseases. New findings also indicate that the treatment of both diseases should include both anticoagulant and anti-inflammatory agents.

## 6. Conclusions

The cardiovascular system, which includes peripheral arterial and venous circulation in addition to the heart, is a complex system with different functional properties. However, the arterial and venous parts of the circulatory system are closely interrelated. This interrelationship is also expressed in pathological states, with inflammation as a basic pathogenetic mechanism of AAD and VTED. Atherosclerosis has already been accepted as a chronic inflammatory disease. However, recent findings indicate the involvement of inflammation in VTED pathogenesis as well. The involvement of inflammation in the pathogenesis of VTED and its relationship to arterial disease is also indicated by increased levels of identical or similar inflammatory markers in subjects at risk for AADs and VTEDs. The recognition of inflammation as a common pathogenetic mechanism of both diseases is especially important from the therapeutic point of view. Recent findings showed that the effects of drugs such as statins also depend on their anti-inflammatory properties. Further, treatment of VTED is based on anticoagulants, but when considering inflammation as a pathogenetic mechanism of VTED, it is expected that anti-inflammatory drugs could also be effective. Preliminary data on the efficacy of new anti-inflammatory drugs showed their utility in the prevention and treatment of both diseases, and they could represent a unified therapeutic approach for the management of AAD and VTED.

## Figures and Tables

**Table 1:** Simultaneous occurrence of venous thromboembolic and arterial atherosclerotic diseases.

Study/Ref	Study/Ref	Findings
Incidence of CV events in pts. with idiopathic VTE [59]	151 pts. with VTE 151 controls	In 43-month follow-up, more CV events in pts. than in controls (16 vs. 6)
CV events after PE [61]	209 pts. with unprovoked PE 105 pts. with provoked PE	In 38-month follow-up, 7.5% CV events in unprovoked PE vs. 3.1% in provoked PE
VTE and subsequent hospitalization due to CV events [63]	20-year cohort study—40,000 pts. with VTE	In the 1st year, relative risk of 1.6 for MI and 2.19 for stroke
Tromso study: risk of stroke in pts. with VTE [64]	82,000 subjects without VTE or CV events	In follow-up of 12.2 years, subjects suffering from VTE had 35% higher risk of CV events, especially stroke
PAD and VTE: ABI studies at Mayo Clinic [65]	39,834 subjects, follow-up for VTE in relation to ABI (34 months)	VTE events occurred in 13% of pts. The highest risk of VTE was in pts. with very low ABI
Relationship between VTE and asymptomatic carotid plaques [62]	299 pts. with DVT 150 controls	Pts. with unprovoked VTE had significantly more plaques than controls (47.1% vs. 32.0%)
DVT: carotid and femoral plaques, IMT [63]	49 pts. with unprovoked DVT 48 controls	Significantly thicker IM and higher prevalence of femoral and carotid plaques than in controls
DVT: ED (flow-mediated dilation of brachial artery) [66]	97 patients with DVT	Decreased FMD (indicator of endothelial dysfunction) in pts.

ABI—ankle–brachial index; CV—cardiovascular; DVT—deep venous thrombosis; ED—endothelial dysfunction; FMD—flow-mediated dilation; IM—intima–media; IMT—intima–media thickness; MI—myocardial infarction; PAD—peripheral arterial disease; PE—pulmonary embolism; VTE—venous thromboembolism; pts.—patients.

## Data Availability

No new data were created or analyzed in this study.

## Abbreviations

AAD	Arterial atherosclerotic disease
ABI	Ankle–brachial index
AMI	Acute myocardial infarction
BMI	Body mass index
CHD	Coronary heart disease
CRP	C-reactive protein
CV	Cardiovascular
DVT	Deep venous thrombosis
ED	Endothelial dysfunction
FMD	Flow-mediated dilation
HDL	High-density lipoprotein
hsCRP	High-sensitivity C-reactive protein
IL-1 beta	Interleukin-1β
IL-6	Interleukin-6
IL-8	Interleukin-8
IL-9	Interleukin-9
IL-10	Interleukin-10
IL-17	Interleukin-17
IM	Intima–media
IMT	Intima–media thickness
LDL	Low-density lipoprotein
MCP-1	Monocyte chemoattractant protein-1
MI	Myocardial infarction
MicroRNA	Micro ribonucleic acid
PAD	Peripheral arterial disease
PE	Pulmonary embolism
TNF-α	Tumor necrosis factor alpha
VT	Venous thrombosis
VTE	Venous thromboembolism
VTED	Venous thromboembolic disease
